# Detection and discovery of plant viruses in *Disporopsis* through high-throughput sequencing

**DOI:** 10.3389/fmicb.2024.1434554

**Published:** 2024-11-13

**Authors:** Qiannan Li, Lianfu Yang, Ting Zhu, Xiyv Yv, Boxin Zhang, Hongzhe Li, Junjie Hao, Lei Zhang, Pengzhang Ji, Jiahong Dong

**Affiliations:** ^1^Institute of Medicinal Plant Cultivation, Academy of Southern Medicine, Yunnan University of Chinese Medicine, Kunming, China; ^2^Yunnan Key Laboratory of Southern Medicinal Resource, School of Chinese Materia Medica, Yunnan University of Chinese Medicine, Kunming, China

**Keywords:** *Disporopsis*, high-throughput sequencing, virome, potyvirus, lispivirus, partitivirus

## Abstract

**Background:**

*Disporopsis*, a member of the *Liliaceae* family and a perennial herb, is predominantly cultivated in southwestern and southeastern China. Its rhizome, referred to as Zhugenqi, serves as a traditional Chinese medicinal herb for the treatment of bone injuries. However, viral diseases have emerged as a significant challenge in the cultivation of *Disporopsis*.

**Objective:**

The aim of this study was to identify and characterize viruses present in diseased samples of *Disporopsis* spp. using high-throughput sequencing (HTS) and reverse transcription-polymerase chain reaction (RT-PCR) to enhance the understanding of the virome associated with *Disporopsis* and to inform diagnostic and control strategies for viral diseases in this plant.

**Methods:**

Diseased samples of *Disporopsis* spp. were subjected to HTS and RT-PCR for virus identification. A total of five viruses were detected, including three novel viruses and two known viruses. The novel viruses were provisionally named *Disporopsis* chlorotic stripe virus (DCSV), *Disporopsis* pernyi-associated partitivirus (DaPTV), and *Disporopsis pernyi*-associated lispi-like virus (DaLV). Sequence identity and phylogenetic analyses were performed to confirm the novelty and taxonomic placement of these viruses.

**Results:**

DCSV exhibited polyprotein sequence identities ranging from 47.6% to 83.6% with other potyviruses, with the highest identity (83.6%) shared with *Polygonatum kingianum* virus 5 (PKgV5). DaLV shared an amino acid sequence identity of 34.59% with maize suscal virus (MSV), and DaPTV shared an identity of 76.18–85.10% with Paris alphapartitivirus (ParAPV). Phylogenetic analyses supported the potential classification of the three novel viruses as new members of their respective genera. Two isolates of polygonatum mosaic-associated virus 1 (PMaV1) were identified in *Disporopsis* for the first time, showing divergences of 96.33% and 98.86% from existing isolates. RT-PCR analysis of 67 *Disporopsis* field samples collected from four cities in China revealed that more than half of the samples tested positive for at least one of the five viruses. PMaV1 and DaLV were the most prevalent, detected in 22 and 34 out of the 67 samples, respectively. Other viruses were detected at low rates and/or had limited distribution.

**Conclusion:**

This study provides insights into the virome infecting *Disporopsis* and offers valuable information for the diagnosis and control of viral diseases in this plant. The identification of five viruses, including three potential new members of their respective genera, contributes to the understanding of the viral threats to *Disporopsis* cultivation.

## 1 Introduction

Plants belonging to be genus *Disporopsis* Hance mainly grow in China and several countries in Southeast Asia such as Laos, Myanmar, Thailand and Vietnam (Ghorbani et al., 2011; Sydara et al., 2014; Nguyen et al., 2019; Sarapan et al., 2023). The rhizome or the entire plant is used as a traditional remedy for easing thirst, nourishing Yin, and moisturizing the body by minority groups in southwest China (Long and Li, 2004; Zeng et al., 2023). Pharmacological studies have shown that the rhizome of *Disporopsis* spp. is rich in bioactive substances such as steroidal saponins, rutin, luteolin, quercetin, and betulinic acid, which have antioxidant, antimicrobial, anti-inflammatory, antibiosis, and anticancer properties (Lin et al., 2014; Wang et al., 2015). The rhizome extracts also have biological control effects on *Pseudoperonospora cubensis*, *Phytophthora infestans* and *Podosphaera xanthii* (Zhu et al., 2018; Han et al., 2022).

High-throughput sequencing (HTS), or deep sequencing techniques, have significantly helped to advances in discovery, diagnostics, and evolutionary studies in virosphere (Roossinck et al., 2015; Roossinck, 2017; Bernardo et al., 2018; Zhang et al., 2018). Many novel and known viruses have been identified in plants, including both wild and cultivated varieties, through the use of high-throughput sequencing (HTS). This is particularly true for medicinal plants such as wild citrus (Kreuze et al., 2009; Zhang and Wang, 2020), greenhood orchids (Pterostylidinae) (Bolger et al., 2014), *Paris polyphylla* (Grabherr et al., 2011), *Mallotus japonicus* (Chen and Chen, 2002), and *Aconitum carmichaelii* (Yang, 2022). The obtained comprehensive genomic data has enabled the virus’ evolution, phytopathology, and epidemiology, as well as disease management strategies, to be further studied (Kreuze et al., 2009).

With the development and industrialization of Chinese herbal medicine, the *Disporopsis* planting areas have been expanded. However, plant diseases have become an important bottleneck to *Disporopsis* production (Zhang and Wang, 2020). In recent years, *Disporopsis* plant leaves displaying virus-like disease symptoms, such as chlorosis, leaf rolling and yellowing, have also been discovered during field surveys. However, there have not yet been any reports about *Disporopsis*-infecting viruses. In this study, we used HTS in combination with RT-PCR to identify *Disporopsis*-infecting viruses in China. Three novel *Disporopsis*-infecting viruses belonging to the genera *Potyvirus*, *Alphapartivirus* and *Lispiviridae*, two different isolates belonging to polygonatum mosaic-associated virus 1 and one isolate of Paris mosaic necrosis virus were identified and characterized. The resulting virus genome information for *Disporopsis* plants have also been made available for future investigations on the evolution, genetic diversity and epidemiology of *Disporopsis*-infecting viruses.

## 2 Materials and methods

### 2.1 Sample collection and field survey

Entire plants of *Disporopsis* were collected from four regions in China, including Kunming city in the Yunnan province, Wenchuan city in the Sichuan province, Enshi city in the Hubei province, and Chongqing city. Additionally, the disease incidence in the field was estimated and recorded.

A total of 67 samples displaying virus-like or unusual symptoms were collected, including 13 *D. pernyi* samples from Kunming, 19 *D. pernyi* samples from Wenchuan, 15 *D. fuscopicta* samples from Enshi, and 20 *Disporopsis* sp. seedling samples (species was not identified) from Chongqing. The *Disporopsis* samples exhibited disease symptoms like leaf stripe, leaf-roll, mottling, and chlorosis. The plants were transplanted into a greenhouse at the Yunnan University of Chinese Medicine for later sampling and testing. Part of the diseased leaves were stored at −80°C. Samples were labeled with the plant species name, symptoms, collection location, and collection date (Supplementary Table S1).

### 2.2 Plant inoculation

Seedlings of *Nicotiana tabacum* var. K326 were obtained through seed germination. Seedlings of *Disporopsis* spp. and *Polygonatum kingianum* were purchased from plantations in Kunming in the Yunnan province and Wenchuan county in the Sichuan province, and they were grown in a growth chamber with a photoperiod of 25°C for 16 h (day)/18°C for 8 h (/night). Mechanical virus inoculation was carried out as follows: the inoculum was prepared by grinding infected leaf tissue with a mortar and pestle in phosphate buffer (mixing 8.0 mL of 1.0 mol-L^−1^ NaH_2_PO_4_ and 42 mL of 1 mol-L^−1^ Na_2_HPO_4_ solution); the leaves of disease-free plants were then lightly dusted with carborundum, and the virus solution was rubbed onto the leaves using a cotton swab. For mock-inoculated control plants, leaves were rubbed with the phosphate buffer alone. The symptomatic and mock-inoculated leaves were then collected at 7 dpi and stored at −80°C for the RT-PCR assay.

### 2.3 High-throughput sequencing and sequence assembly

The samples from Kunming, Yunnan province, exhibiting chlorotic stripe symptoms were sent to OE Biotech Co., Ltd. (Shanghai, China) for HTS. The total RNA was extracted using TRIzol (Ambion, Hillsboro, Oregon, United States) following the manufacturer’s instructions. Ribosomal RNA was depleted using the RiboZero kit (Illumina, San Diego, CA, United States) according to the manufacturer’s instructions. cDNA libraries were synthesized using the TruSeq Stranded Total RNA Kit with Ribo-Zero Gold (Illumina). The obtained cDNA libraries were paired-end deep sequenced reads using the Illumina HiSeq 2500. Raw data (raw reads) were processed using Trimmomatic by removing the reads containing ploy-N and the low-quality reads (Bolger et al., 2014). After removing the adaptor sequences, the clean reads were assembled into expressed sequence tag contigs and *de novo* assembled into transcripts using Trinity 2.4 version in paired-end mode (Grabherr et al., 2011). For subsequent analysis, the longest transcript was designated as an unigene based on the similarity and length of the sequence. The unigenes were functionally annotated by querying them against the NCBI nonredundant (NR), SwissProt, and Clusters of orthologous groups for eukaryotic complete genomes (KOG) databases using Blastx with a threshold *E*-value of 10^−5^.

### 2.4 RT-PCR

The total RNA was extracted using the Eastep^®^ Super Total RNA Extraction Kit according to the manufacturer’s instructions. Specific primers for each virus were designed based on the transcriptome contigs (Table 1) and were then used in RT-PCR to amplify the corresponding viral fragments. RT-PCR was conducted using a PrimeScript^TM^ One-step RT-PCR Kit ver.2 (TaKaRa Biotechnology Co., Ltd., Dalian, China). The 25 μL RT-PCR reaction contained 3 μL of total RNAs, 0.5 μL of each primer (10 μM), 12.5 μL of 2 × 1 Reaction Mix, 1 μL of Enzyme Mix and 7.5 μL of distilled water. The thermal cycling conditions were 1 cycle of reverse transcription at 50°C for 30 min and denaturation at 94°C for 2 min, 35 cycles of amplification at 94°C for 30 s, annealing temperature for 30 s and 72°C for 60 s, and a final extension of 72°C for 10 min. Sanger sequencing of the target fragment clones was subsequently performed to verify the accuracy of the new and known viruses identified by HTS. The complete genome sequence of Polygonatum mosaic virus 1-*Disporopsis* 1 (PMaV1-D1) was obtained by assembling the Sanger sequenced RT-PCR and 5′ RACE amplified reads, and the partial genome sequences of the DCSV, DaLV, and PMaV1-D2 isolates were assembled from the RT-PCR amplified sequenced reads. The viral full-length primers were designed based on the allele sequences of each target virus (new virus or known virus) obtained in this study (Supplementary Tables S2–S5). Based on a modified 5′RACE method by Chen and Chen (2002) and Yang (2022), the first-strand cDNA was first synthesized by reverse transcription using super M-MuLV Reverse Transcriptase from Sangong Bioengineering Co. (Shanghai). The total RNA was converted into cDNA in a 5 μL reaction that consisted of 1 μL total RNA, 1 μL the 3′-specific primer ZHM1, 1 μL of dNTPs, and 2 μL DEPC. The reaction was incubated at 65°C for 5 min and then left on ice for 2 min. Next 2 μL 5 × Super M-MLV Buffer, 1 μL of Solution I, 0.5 μL of RNase Inhibitor, 0.5 μL of Super M-MLV Reverse, and 1 μL of DEPC were mixed with the cDNA solution to form a 10-μL system solution, and the reaction was incubated at 42°C for 60 min and then 80°C for 15 min. Subsequently, a PCR was performed using the cDNA and the 3′-specific primer ZHM1. The 50 μL reaction contained 0.5 μL of cDNA, 0.5 μL of Ex Taq, 0.5 μL of 5′-end specific primers, 0.5 μL of ZHM1, 0.5 μL of dNTPs, and 38 μL of DEPC. The thermal cycling conditions were as follows: 2 min at 94°C, 35 cycles of 30 s at 94°C, 30 s at TM, and 72°C for 60 s, and finally, an extension period of 72°C for 10 min. The products were recovered, cloned and Sanger sequenced as described above.

**Table 1:** Primers used for detection of plant viruses in *Disporopsis*.

Primers name	Primer sequences	Amplification length (bp)	TM (°C)
DCSV-CP-F	CATTGAATCGTGGGGTTATGACAAGTTGA	1,088	62
DCSV-CP-R	CGGATACGAGGTAAAACCTCACCACACC		
PMaV1-Df1-4356F	GAAGAGAGTGCGAGTTCAACACGCAG	1,457	64
PMaV1-Df1-5813R	GTGCATTCGCTTCTTGCCTTGGTATG		
PMaV1-Df2-4181F	GACGAGTGGCTTCGCGTTGCATTAC	1,518	60
PMaV1-Df2-5699R	TGAGACTAAGCTATTGTTCCAGCGCCC		
PMNV-CP-F	CCTTCAACAGTTGTGGGACAACACACT	1,400	60
PMNV-CP-R	AACGACGCGAGATGCTAACTGT		
DaLV-4669F	TGGACATGCTTGATGACTTCTCTGA	591	60
DaLV-5260R	CAGTCATTGTCATACTGCCGAATGTC		
DaPTV-RNA1-F	GGAAAAGATTCGACAAAGATGCAC	841	58
DaPTV-RNA1-R	GCCAGTGCTTCTCAGAGAGTAGC		
DaPTV-RNA2-F	TCCGCGGCCAGCTTCTTAGAGGGAC	1,688	59
DaPTV-RNA2-R	GAGATCAGAT CCCCGTTCGT GGGGTCC		

### 2.5 Sequence analysis

The sequence identities of the isolate viruses were analyzed alongside sequences of published viruses in the NCBI database using DNAMAN vers.7.0 (LynnonBiosoft, United States). Open reading frames (ORFs) were predicted using ORF Finder on the NCBI website.^1^ The phylogenetic relationships among the amino acid sequences were analyzed in Mega X. The phylogenetic trees were constructed using the maximum likelihood algorithm and LG model with 1,000 bootstrap replicates (Kumar et al., 2018).

## 3 Results

### 3.1 Symptoms of virus-infected *Disporopsis*

**Figure 1:**
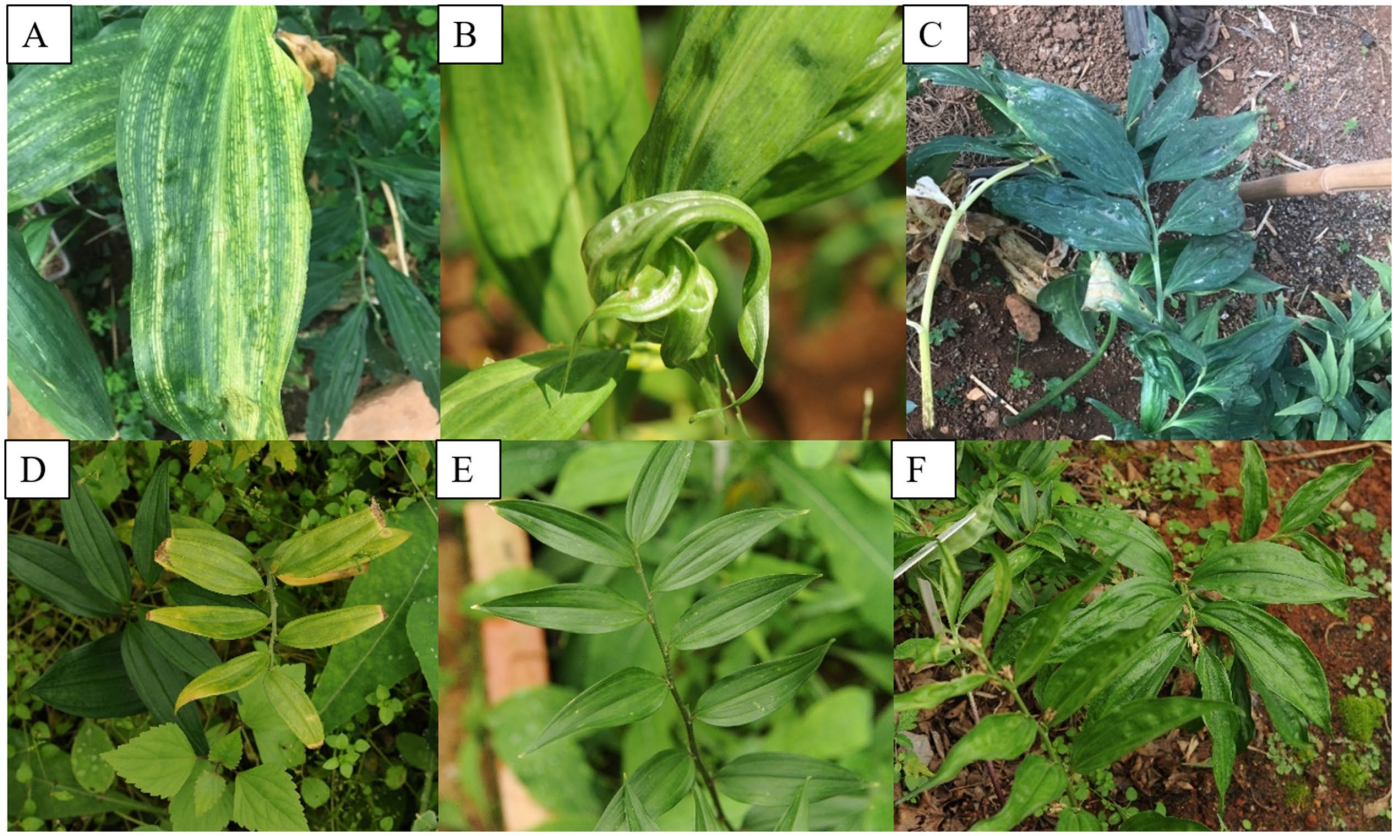
Typical symptoms of virus diseases on leaves of *Disporopsis* plants: (A) chlorotic stripe, (B) leafroll, rugose and mottle, (C) mottle, (D) yellow, (E) mild mottle, (F) rugose. (A,B) *D. longifolia* from Yunnan Province, (C–F).

The virus-infected *Disoropsis* plants displayed the following symptoms: chlorotic stripes along the leaf vein, top leaf rolling, and mottle and mosaic symptoms on leaves (Figure 1). Four viruses except DaPTV were detected in (A), DSCV and DpLV were detected in (B), all viruses were detected in (C), DpLV and DaPTV were detected in (D,E) and DCSV and PMaV1-D1 were detected in (F). The most severe symptoms appeared between June and September in Kunming. No symptoms were observed on healthy plants.

### 3.2 Identification of viruses found in *Disporopsis pernyi* using HTS

HTS was performed on the leaves from one *Disporopsis pernyi* plant that showed chlorotic stripe symptoms in Kunming (E102°49′16″, N24°50′9″). A total of 24.28 M raw reads were obtained, with a total length of 2,201,943 bp and an average GC content of 61.14%. After quality trimming and size filtering, approximately 24.06 M clean reads remained. Subsequently, the clean reads were *de novo* assembled into 3,532 contigs and annotated by using BLASTx to search the GenBank database and identify the potential viral agents. Next, seven contigs were mapped to viral genome sequences in GenBank (Table 2). One 9,685 nucleotide long contig had a high amino acid sequence identity of 82% with *Polygonatum kingianum* virus 5 (GenBank accession number: MN873571.1), which belonged to the genus *Potyvirus*. Two of 9,635 nt and 8,863 nt contigs had high aa sequence identities of more than 93% with polygonatum mosaic-associated virus (PMaV1, GenBank accession number: OP380926.1), One contig of 7,531 nt in length had a high amino acid sequence identity of 34.59% with the ORF4-encoded protein of maize suscal virus (GenBank accession number: MZ270532.1), which belonged to the *Lispiviridae* family. Lastly, two contigs of 2010 nt and 1873 nt in length had high amino acid sequence identities with RdRp and CP of Paris alphapartitivirus (GenBank accession number: OL960006.1 and OL960007.1). RT-PCR assays and Sanger sequencing further confirmed the HTS results. The viruses in the HTS-tested *D. pernyi* included three novel viruses and two PMaV1 isolates. Three novel viruses were temporarily named *Disporopsis* chlorotic stripe virus (DCSV), *Disporopsis pernyi* associated partitivirus (DaPTV) and *Disporopsis pernyi* associated lispi-like virus (DaLV), respectively.

**Table 2:** Main viruses identified through high through sequencing.

Contigs ID	Virus name	Amino acid Identity	GenBank ID
NODE_7_length_9683_cov_904.613263	*Polygonatum kingianum* virus 5	82.56%	QVN46485.1
NODE_8_length_9635_cov_75.730383	Polygonatum mosaic-associated virus 1	96.33%	OP380926.1
NODE_9_length_8868_cov_22.325219	Polygonatum mosaic-associated virus 1	93.12%	OP380926.1
NODE_10_length_7531_cov_26.189697	Maize suscal virus	34.59%	UWX11517.1
NODE_423_length_2010_cov_1294.097775	Paris alphapartivirus 1	85.10%	UXX19573.1
NODE_514_length_1873_cov_52.519488	Paris alphapartivirus 1	75.76%	UXX19572.1
NODE_7904_length_155_cov_58.948718	Paris alphapartivirus 1	79.40%	UXX19572.1

### 3.3 Characterization and analysis of the complete genomic sequence of *Disporopsis* chlorotic stripe virus, a novel potyvirus

**Figure 2:**
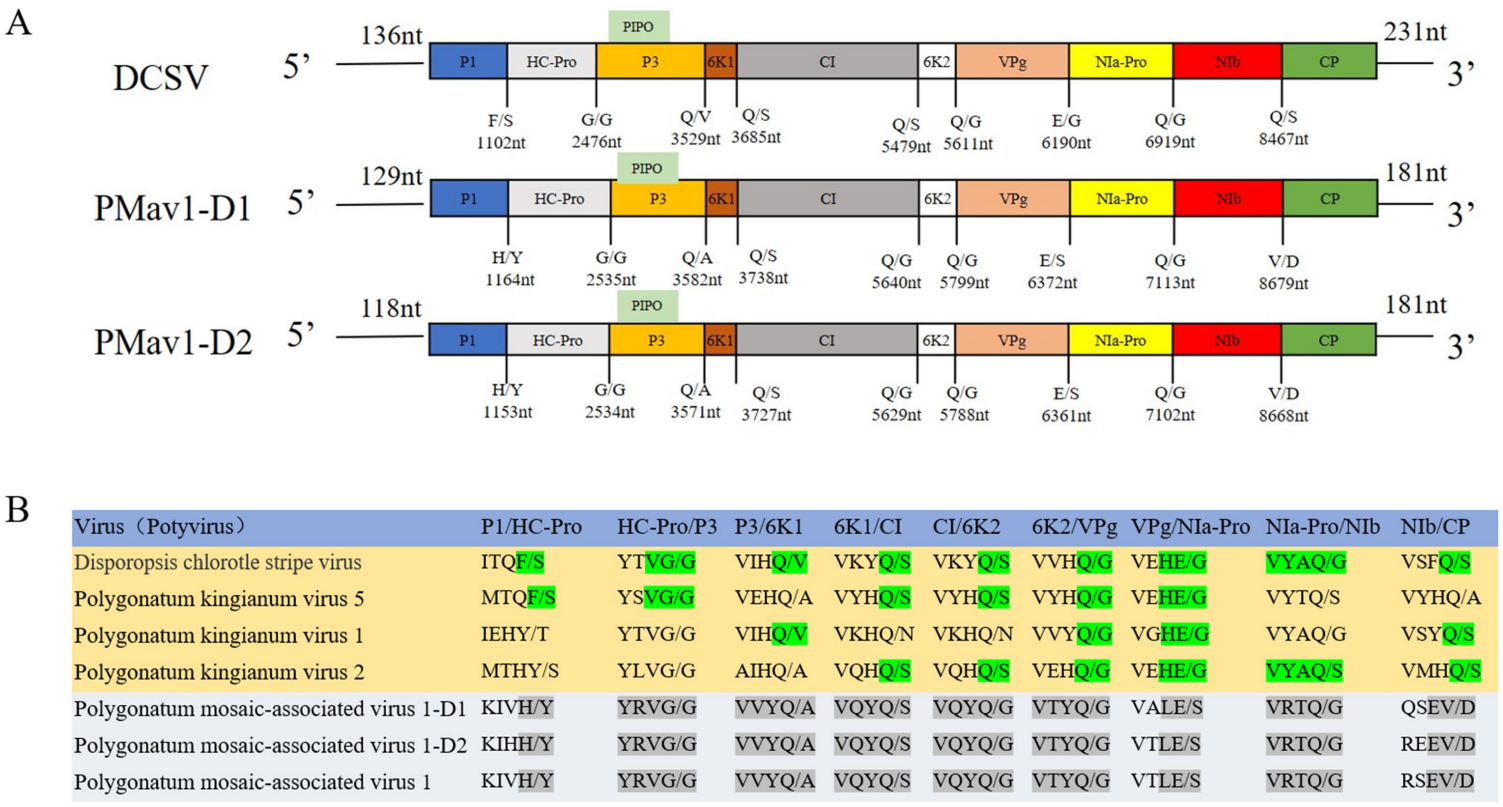
**(A)** The cleavage sites of DCSV, PMaV1-D1, and PMaV1-D2, along with their corresponding proteins. These sites were sequentially cleaved into nine mature proteins, including P1, HC-Pro, P3, 6K1, CI, 6K2, VPg, NIa-Pro, NIb, CP. **(B)** PMaV1 isolates protein cleavage sites and corresponding proteins.

The near complete genomic sequences of *Disporopsis* chlorotic stripe virus (DCSV, GenBank accession number: PP691760) was determined to be 9,640 nt long using RT-PCR, excluding the 3′-terminal poly(A) tail. The genome organization of DCSV was identical to that of other members of the *Potyvirus* genus in the *Potyviridae* family and contained a large ORF encoding a polyprotein of 3,090 aa residues. The 5′ and 3′ untranslated regions (UTR) were 136 nt and 231 nt long, respectively. Nine highly conserved proteolytic cleavage sites in the polyprotein were identified by comparing them with the consensus protease recognition motifs from other potyviruses. Ten putative mature proteins were identified from these cleavage sites: P1 (321 aa), HC-Pro (458 aa), P3 (351 aa), 6K1 (52 aa), CI (589 aa), 6K2 (53 aa), VPg (193 aa), NIa-Pro (243 aa), NIb (516 aa) and CP (314 aa) (Figure 2). The small ORF PIPO within the P3 of potyviruses was also identified due to the presence of GAAAAAAA in the DCSV genome sequence (2,932–2,939 nt).

**Figure 3:**
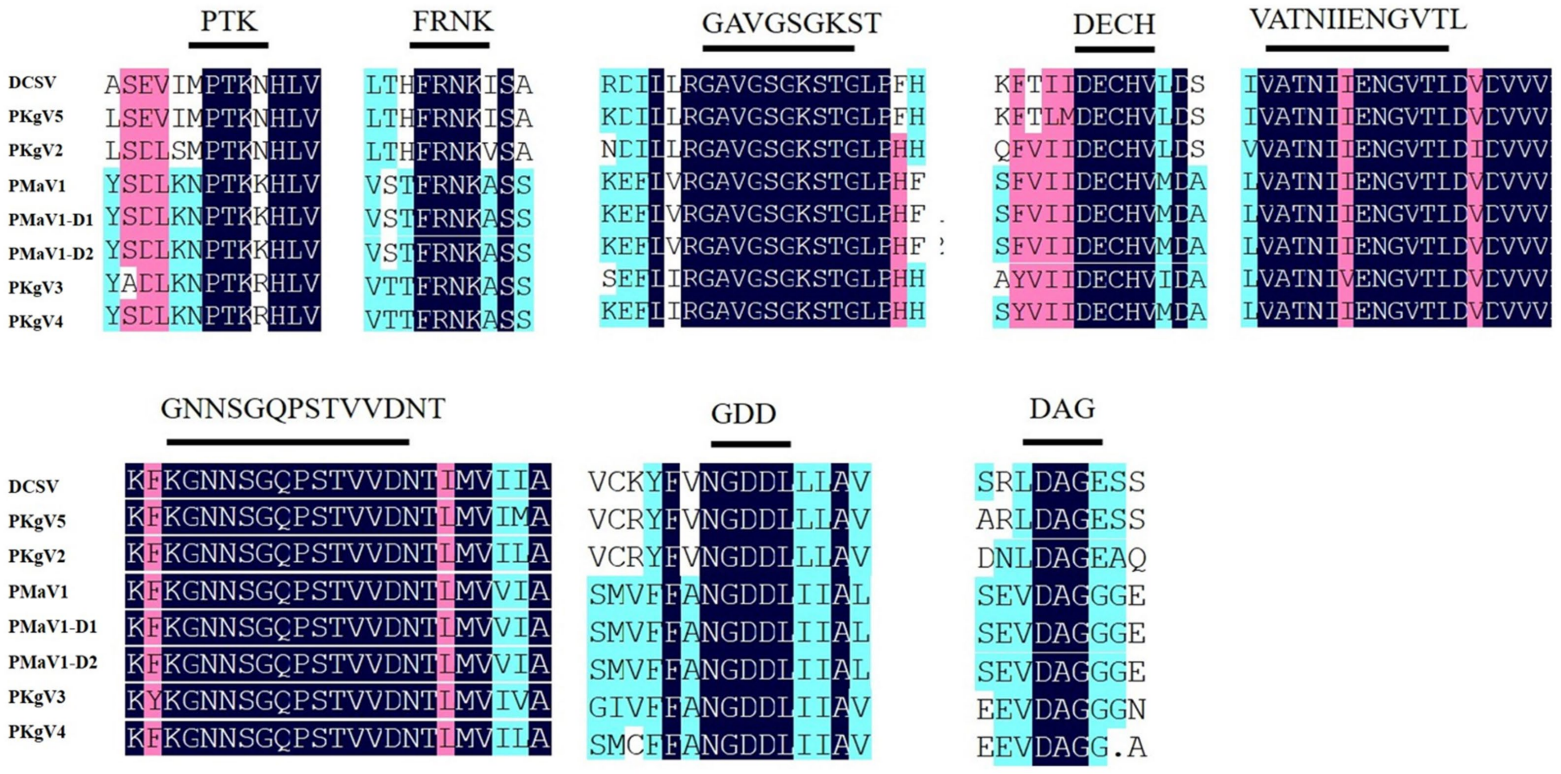
Conserved motifs among DCSV, PMaV1-D1, PMaV1-D2 and other potyviruses. These polyproteins contain the typical highly conserved potyviral motifs. The shaded black areas indicate conserved amino acid residues, respectively.

Furthermore, eight highly conserved motifs were found in the polyprotein amino acid sequences. The high level of conservation among these sequences demonstrated that these proteins likely perform essential functions in viruses and therefore are retained over the course of viral evolution (Zhang et al., 2023). These include the ^630^PTK^632^ (aphid transmission) and ^501^FRNK^504^ motifs in HC-Pro; ^1266^GAVGSGKST^1274^ (NTP binding), ^1355^DECH^1358^ (potential helicase activity), and ^1484^VATNIIENGVTL^1495^ (potential helicase activity) motifs in CI, ^2612^GNNSGQPSTVVDNT^2625^ (RNA-dependent polymerase activity) and ^2656^GDD^2658^ (RNA-dependent polymerase activity) motifs in NIb, and ^2858^DAG^2860^ (aphid transmission) motif in CP (Figure 3) (Valli et al., 2007; Puli’uvea et al., 2017; Worrall et al., 2019; Zheng et al., 2008; Chung et al., 2008). The numbers in superscript represent the positions of the sequences along the DCSV genome.

The genome sequences of DCSV and other potyviruses listed in Supplementary Tables S6, S7, S10, S11, except for PKgV5, shared nucleotide identities of 53.10–69.60% and amino acid identities of 48.10–75.70%. Five proteins, P1 (14.10–54.00%), HC-Pro (46.30–71.10%), P3 (25.50–64.10%) and 6K2 (41.50–58.50%), VPg (51.90–79.40%), had the amino acid identities of less than 80% with other potyviruses, other five proteins, 6K1 (42.30–82.70%), CI (55.70–80.80%), NIa-Pro (52.70–83.10%), NIb (58.30–83.40%) and CP (59.80–86.90%) had amino acid identities of more than 80% with other potyviruses. These values were all under the current species demarcation threshold (Supplementary Tables S6, S8) for the *Potyviridae* family (Shukla and Ward, 1989; Adams et al., 2005). Sequence alignment comparisons showed that the polyprotein and CP of DCSV had more than 82% amino acid identity with PKgV5, which is beyond the current species demarcation threshold (Supplementary Table S6). However, given that the genome sequence of PKgV5 was only confirmed using the assembled HTS contig data, without using RT-PCR, we propose that only DSCV is a novel potyvirus, and name it *Disporopsis* chlorotic stripe virus.

**Figure 4:**
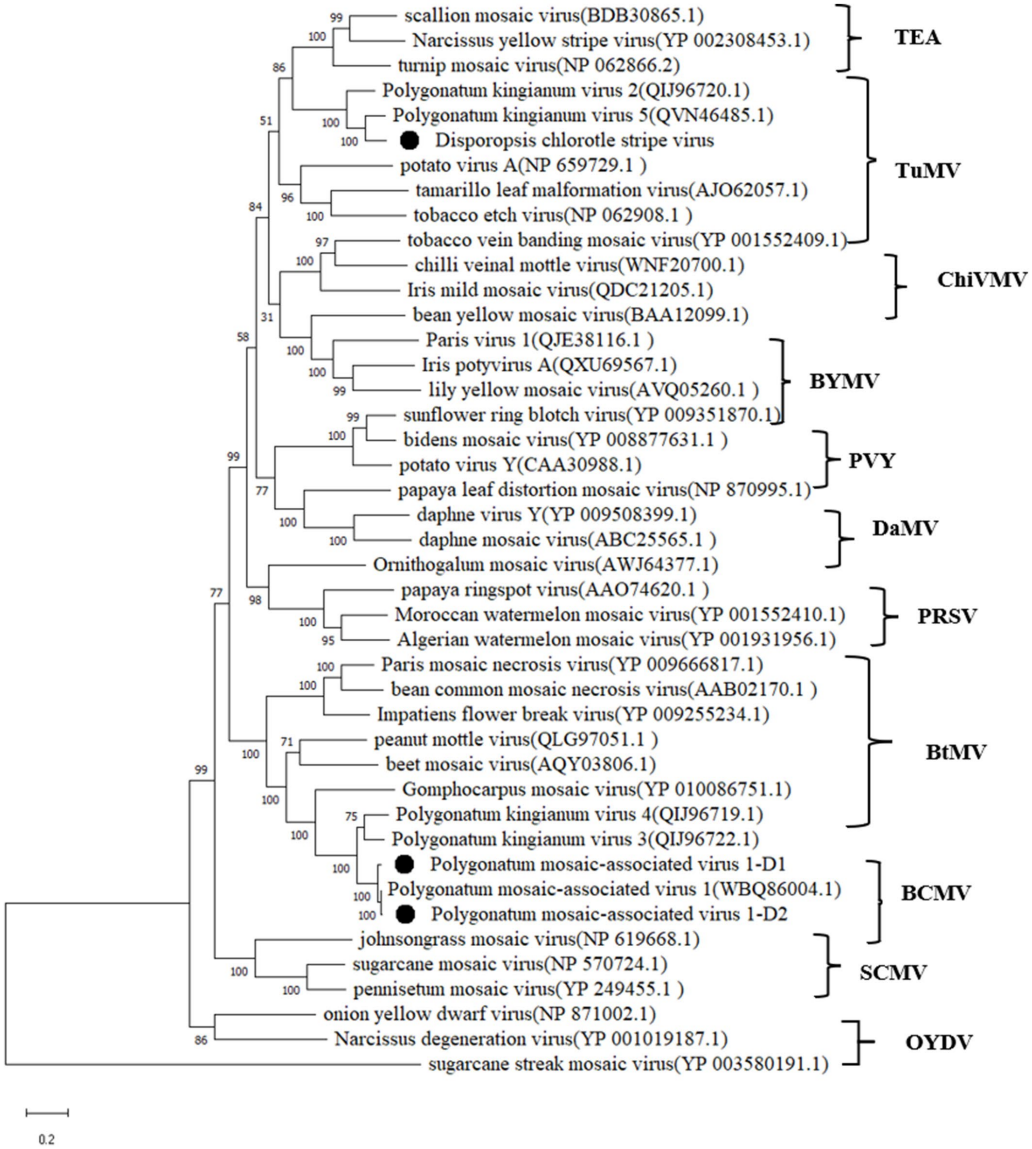
Maximum-likelihood tree based on the deduced polyprotein sequences of DCSV, PMaV1-D1, PMaV1-D2 and representative members of the genus *Potyvirus*. Each of the subgroups is indicated by abbreviation of the representative virus. Bootstrap analysis was applied using 1,000 bootstrap replicates. The scale bar representing a genetic distance of 0.2. Sugarcane streak mosaic virus, a member of the genus *Poacevirus*, was used as an outgroup.

The phylogenetic analyses were performed using the deduced polyprotein sequences of DCSV and 42 other potyviruses available in GenBank (Figure 4). The maximum likelihood tree placed DCSV within the *Potyvirus* genus and into the TuMV subgroup. DCSV was most closely related to putative polyprotein sequences belonging to an unconfirmed *Polygonatum kingianum* virus 5 (PKgV5, QVN46485.1) and *Polygonatum kingianum* virus 2 (QIJ96720.1).

### 3.4 Sequence analyses of two PMaV1 isolates

PMaV1 is a potyvirus that was first identified in *Polygonatum cyrtonema* Hua (Li et al., 2023). In this study, two PMaV1 isolates were detected in *Disporopsis*, and were named PMaV1-*Disporopsis* 1 (GenBank accession number: PP691759) and PMaV1-*Disporopsis* 2 (GenBank accession number: PP691758), respectively. The complete genome sequences were determined to be 9,637 nt and 9,626 nt in length, excluding the 3′-terminal poly(A) tail, using RT-PCR. PMaV1-*Disporopsis* 1 and PMaV1-*Disporopsis* 2 encoded a polyprotein of 3,109 aa and 3,084 aa, respectively. The 5′and 3′-UTRs of PMaV1-*Disporopsis* 1 were 129 nt and 181 nt. The 5′ and 3′-UTRs of PMaV1-*Disporopsis* 2 were 118 nt and 181 nt (Figure 2). The polyproteins of the two PMaV1 isolates had 96.33 and 98.86% identity with those of a PMaV1 isolated from *Polygonatum cyrtonema*, respectively. All three PMaV1 isolates shared the same conserved motifs (Li et al., 2023). The size difference in the polyproteins of the two *Disporopsis* isolates of PMaV1 was due to the deletion of the CP N-terminal. The CP of PMaV1-*Disporopsis* 1 and *Disporopsis* 2 consisted of 260 aa and 183aa residues, respectively (Supplementary Tables S8, S10, S11). Nine other proteins within the two isolates were identical in size (Figure 2). Moreover, PIPO ORF was identified due to the presence of GAAAAAA in PMaV1-*Disporopsis* 1 (nt positions 2,992–2,998) and PMaV1-*Disporopsis* 2 (nt positions 2,981–2,987), respectively (Figure 2).

### 3.5 PMNV detection

Paris mosaic necrosis virus (a potyvirus) was first reported in *Paris polyphylla* var. *yunnanensis*, which belongs to the *Liliaceae* family, and subsequently detected in *Polygonatum kingianum* (Lan et al., 2018; Hu et al., 2022). PMNV was also detected in four *D. pernyi* samples from Kunming using RT-PCR with the virus specific primer pairs targeting NIb, CP, and the 3′-UTR sequence of PMNV. The RT-PCR products were cloned and sequenced. Sequence analysis showed that the CP gene of the four PMNV *Disporopsis* isolates was 813 nt long and shared 86.4–92.4% and 90.4–95.8% of their nucleotide and amino acid sequences, respectively, with PMNV-cn isolate from *P. polyphylla* var. *yunnanensis*.

### 3.6 RdRp sequence analysis of a lispi-like virus

**Figure 5:**
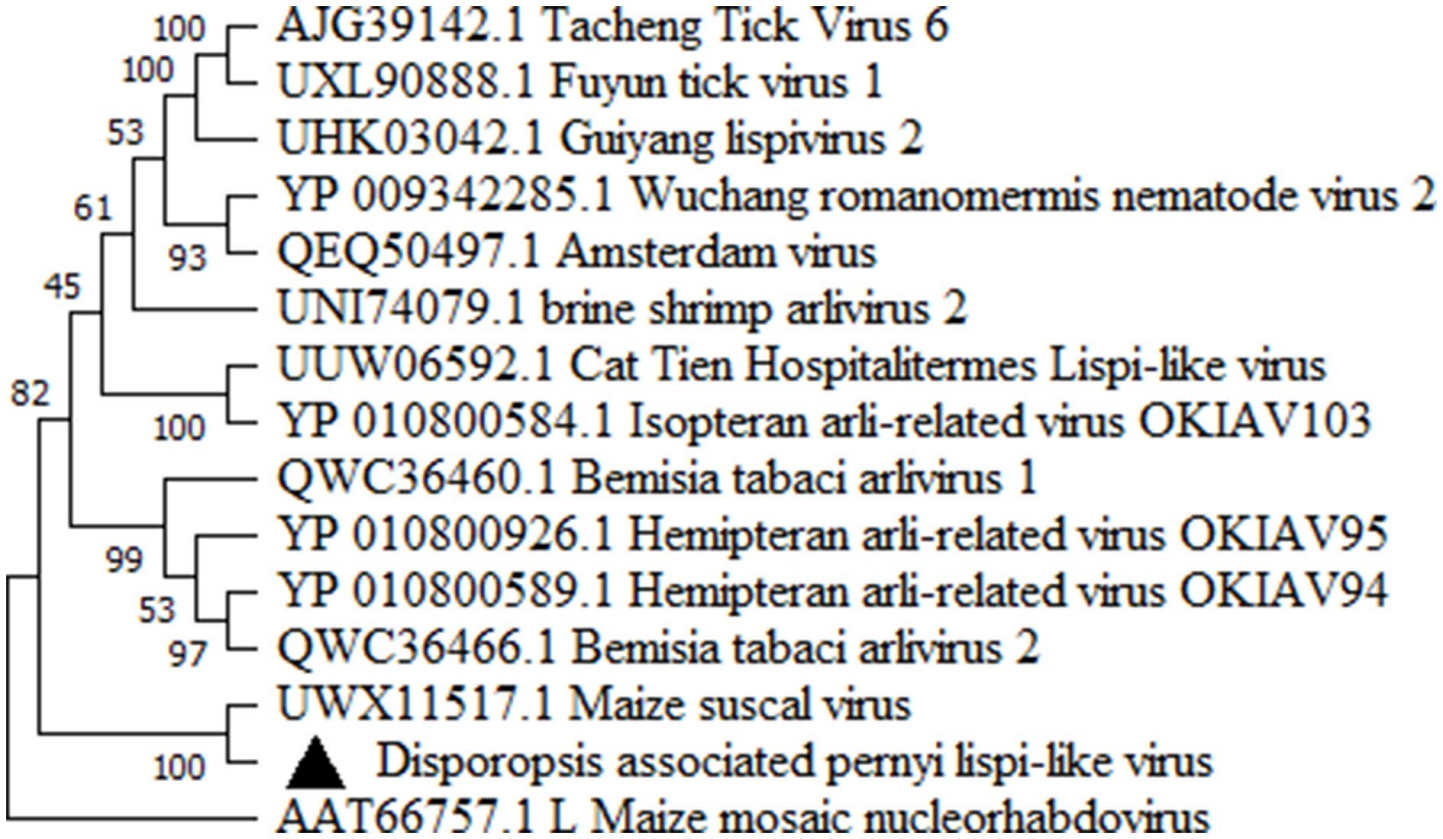
Maximum-likelihood tree based on the deduced polyprotein sequences of *Disporopsis pernyi* lispi-like virus representative members of the family *Lispiviridae*. Each of the subgroups is indicated by abbreviation of the representative virus. Bootstrap analysis was applied using 1,000 bootstrap replicates. The scale bar representing a genetic distance of 0.5.

A 7,531 nt contig was highly homologous to the RdRp of maize suscal virus (GenBank accession number: MZ270532), with a query coverage of 80.00% and an amino acid identity of 34.59%, as well as to other lispi-like viruses with a query coverage of about 50.00% and amino acid identity of about 28% (Supplementary Table S9). Sequence analysis showed that the RdRP of *Disporopsis pernyi*-associated lispi-like virus possessed the conserved motifs: *Mononegavirales* RNA-dependent RNA polymerase (pfam00946) and *Mononegavirales* mRNA-capping region V (pfam14318) using NCBI Conserved Domain Search^2^ (Wang et al., 2023). The phylogenetic tree based on the RdRP from lispi-like viruses showed that *Disporopsis pernyi* associated lispi-like virus was clustered in a subbranch with maize suscal virus (Figure 5). Using the specific primer pairs derived from this contig (Supplementary Figure S1), we detected this lispi-like virus in 32 *Disporopsis* samples using RT-PCR, there by indicating that this virus (putatively named *Disporopsis pernyi* lispi-like virus, GenBank accession number: PP803564) occurred frequently in *Disporopsis*.

### 3.7 *Disporopsis pernyi* associated partitivirus

**Figure 6:**
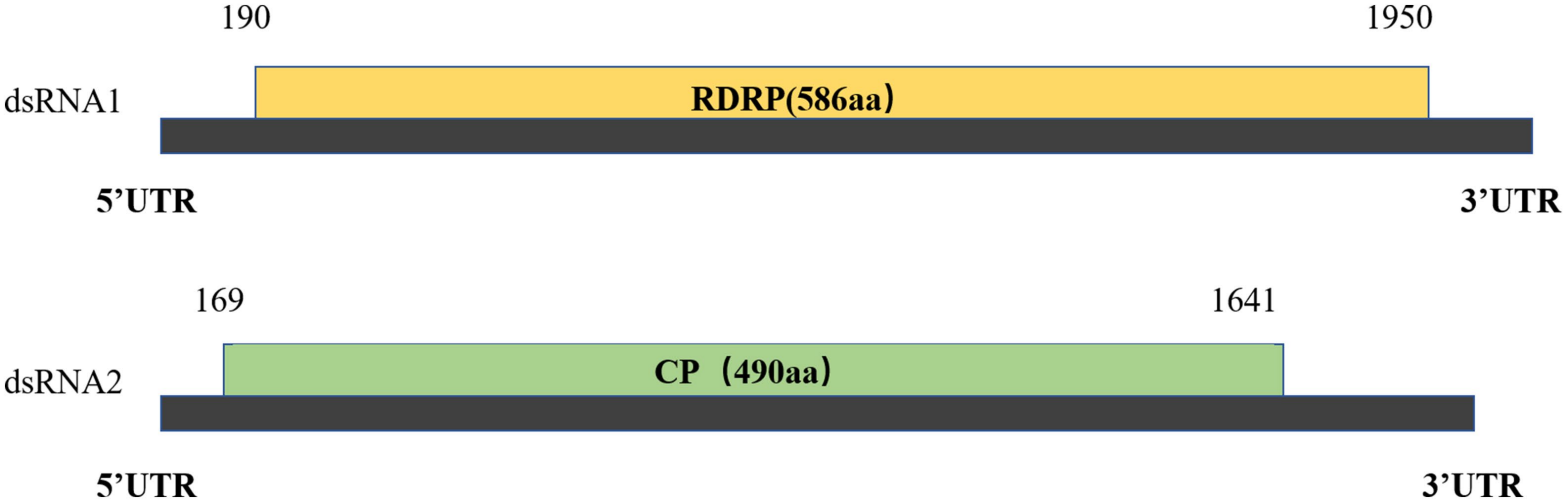
Schematic representation of DpPV dsRNA1 and dsRNA2 with ORFs indicated.

**Figure 7:**
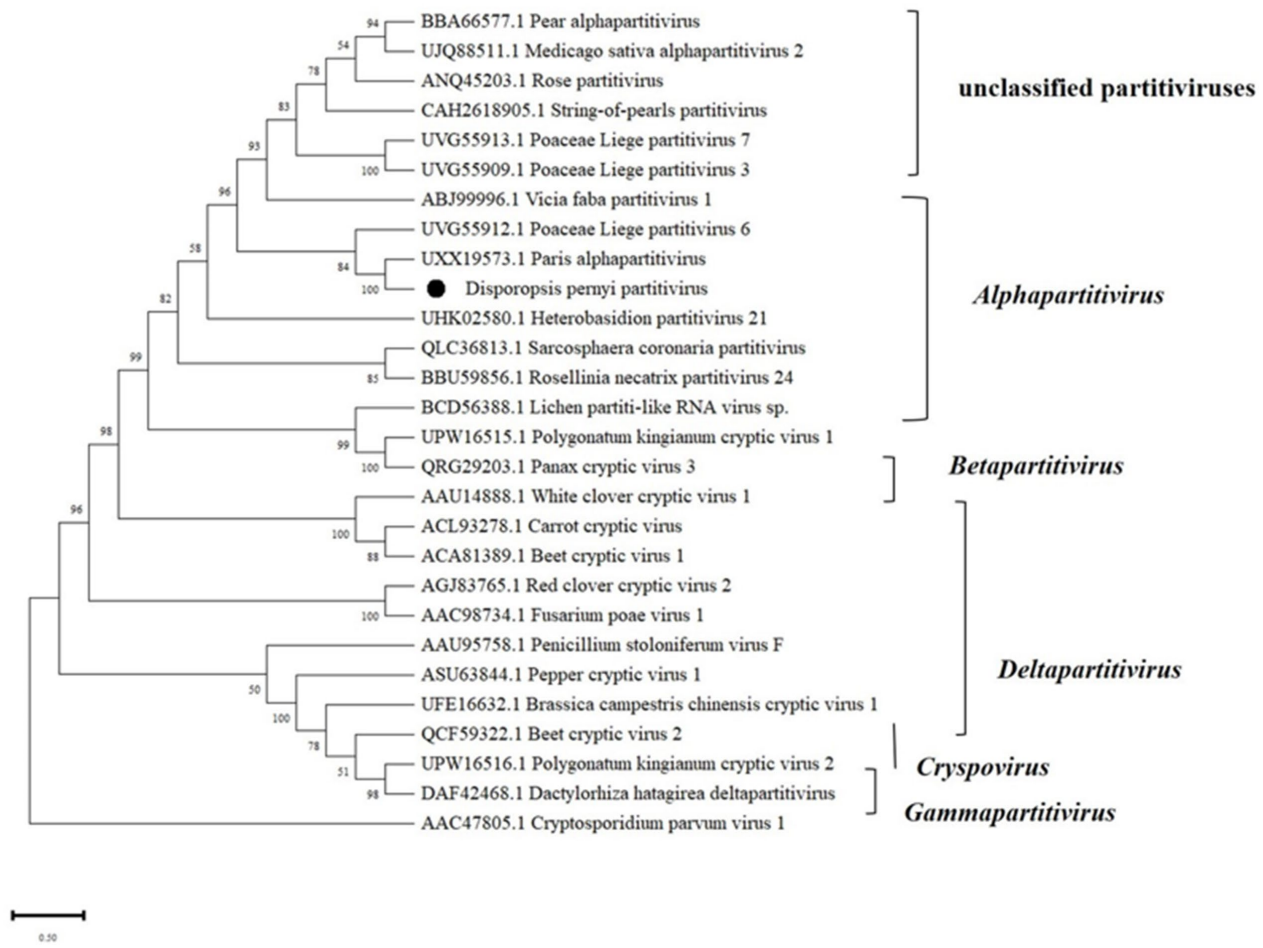
Maximum likelihood phylogenetic tree of amino acid sequences of the RdRp of representative members of the family *Partitiviridae*. Bootstrap probabilities for each branch node were estimated using 1,000 replicates. Phylogenetic tree was constructed using MEGA X software. *Cryptosporidium parvum* virus 1 was used as outgroup. Viral genome sequences were downloaded from NCBI viral genome database (http://www.ncbi.nlm.nih.gov/genome/viruses/). The scale bar representing a genetic distance of 0.5.

**Figure 8:**
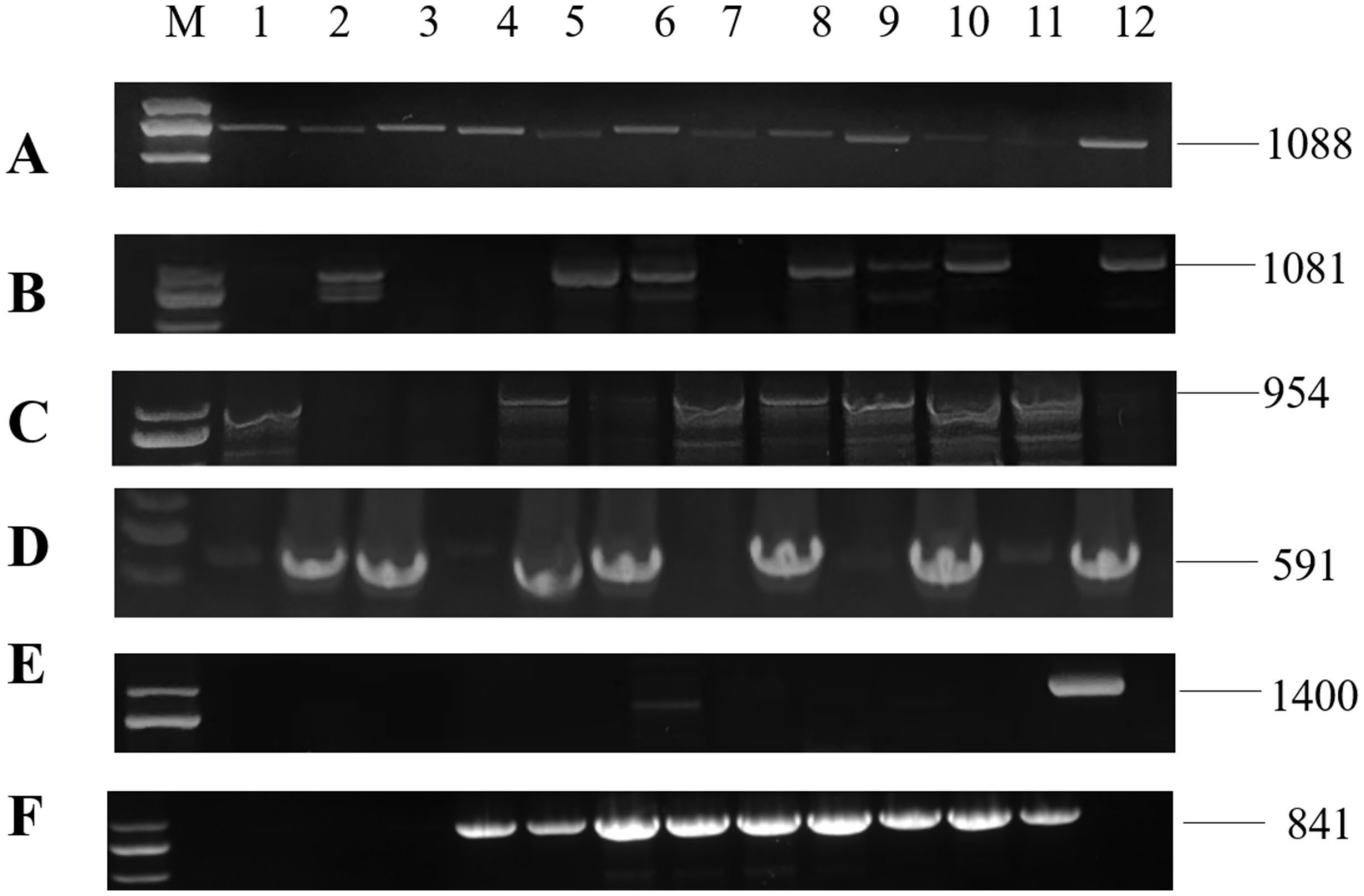
RT-PCR detection for viruses in diseased plants of *Disporopsis*: (A) DCSV, (B) PMaV1-D1, (C) PMaV1-D2, (D) DpLV, (E) PMNV, (F) DaPTV, Lanes (M) DNA marker, (D-KM1-12) diseased samples.

RNA1 and RNA2 of *Disporopsis pernyi*-associated partitivirus (DaPTV, GenBank accession number: PP803563 and PP803565) were determined to be 2,010 nt and 1,873 nt in length. The genome structure of DaPTV was identical to that of other alphapartitiviruses (Figure 6). RNA1 encoded an RdRp of 586aa (190–1,950 nt) and RNA2 encoded a CP of 490 aa (169–1,641 nt), Both RNA1 and RNA2 had high amino acid sequence identities of 85 and 76.1% with those of Paris alphapartitivirus (GenBank accession number: OL960006.1 and OL960007.1) (Table 3). These percent values were all under the current species demarcation threshold (≤ 90% aa sequence identity in the RdRP, and ≤80% aa sequence identity in the CP) (Vainio et al., 2018) but within the *Alphapartitivirus* genus (Figure 7). Thus, we suggest that DaPTV is a new member of the *Alphapartitivirus* genus. To confirm the presence of the virus in *Disporopsis*, RT-PCR was conducted using specific primer sets (as listed in Table 1) that target RNA1 and RNA2. The sequences obtained from the RT-PCR products were then analyzed and confirmed to match the virus’s identity. DaPTV-RNA1 primers were used to detect 13 samples from Kunming, 9 samples were found to have bands, which proved the existence of DaPTV (Figure 8).

**Table 3:** Amino acid (aa) sequence identities of *Disporopsis pernyi* associated partitivirus and members of the family *Partitiviridae*.

Genus	Virus name	Viral genome sizes (bp)		Encoded protein sizes (aa)		Sequence identity (%) for encoded proteins	
		RdRp	CP	RdRp	CP	RdRp	CP
Alphapartitivirus	Paris alphapartitivirus	1,917	1,818	586	493	85.1	76.18
	String-of-pearls partitivirus	1,927	1,792	586	490	71.4	52.4
	Pear alphapartitivirus	1,945	1,788	586	491	71.23	55
	Poaceae Liege partitivirus 3	1,828	—	579	—	71.16	—
	Poaceae Liege partitivirus 6	1,902	—	586	—	71.06	—
	Medicagosativa alphapartitivirus 2	1,859	1,764	586	491	71.06	53.7
	Medicagosativa alphapartitivirus 1	1,967	1,679	586	499	69.3	60.6
	Rosellinia necatrix partitivirus 2	1,985	1,828	603	483	64.5	40
	Rose partitivirus	1,937	1,811	586	487	73	52.1
	Beet cryptic virus 1	2,008	1,783	616	489	33.4	17.6
	Carrot cryptic virus	1,971	1,776	616	490	32.1	17.6
Betapartitivirus	Ceratocystis resinifera virus 1	2,207	2,305	663	661	14	11.7
	Pleurotus ostreatus virus 1	2,296	2,223	706	636	21.8	11.4
Gammapartitivirus	Ophiostoma partitivirus 1	1,744	1,567	539	430	15.2	13.9
	Penicillium stoloniferum virus F	1,677	1,500	538	420	21.1	8.9
Deltapartitivirus	Fig cryptic virus	1,696	1,415	472	337	18.2	10.7
	Pepper cryptic virus 2	1,609	1,525	478	430	16.2	10.7
Cryspovirus	*Cryptosporidium parvum* virus 1	1,836	1,510	523	319	14.5	11.4

The deeper the red, the higher the similarity; the deeper the blue, the lower the similarity.

### 3.8 Infectivity of DCSV, PMaV1, and PMNV

**Figure 9:**
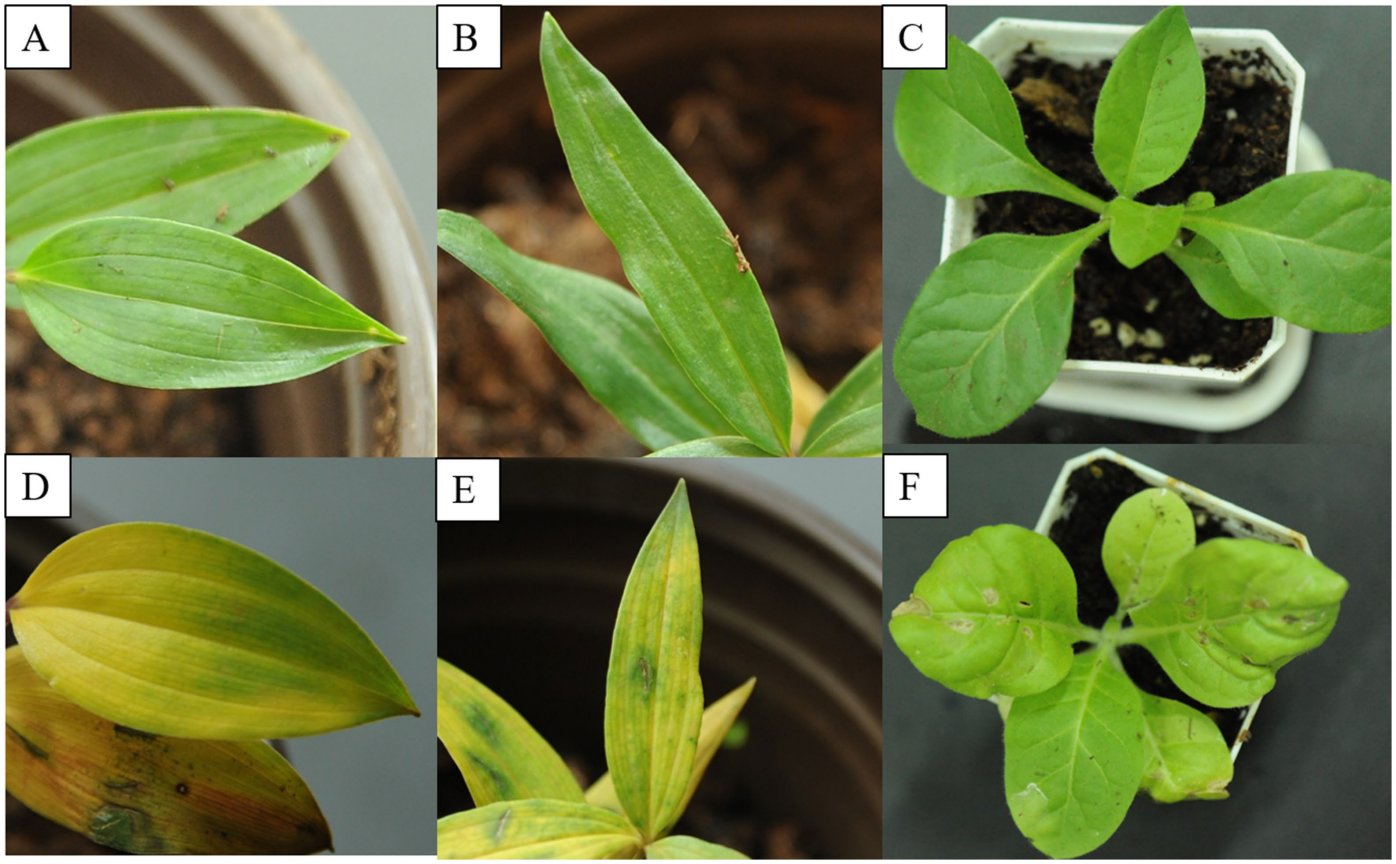
Infectivity of DCSV, PMaV1 and PMNV. (A–C) Represents virus-free seedling of *D. pernyi*, *P. kingianum* and *N. tabacum* var. *K326*, respectively. (D–F) Indicates the symptomatic leaves of *D. pernyi*, *P. kingianum* and *N. tabacum* var. *K326*, respectively.

In order to test the infectivity of DCSV, PMaV1 and PMNV in *Disporopsis*, the viruses were extracted from three virus-infected leaves exhibiting chlorotic and yellow stripe, and mottle symptoms and were mechanically inoculated into virus-free seedlings of *Disporopsis* spp., *Polygonatum kingianum* and *Nicotiana tabacum* var. K326. At 7 days post-inoculation (dpi), the leaves of the inoculated *Disporopsis* plants displayed the characteristic chlorotic stripe symptoms, and DCSV, PMaV1, and PMNV were detected using RT-PCR. The leaves of the *P. kingianum* plants displayed the characteristic yellow and necrotic spot symptoms, and DCSV and PMaV1 were detected using RT-PCR (Figure 9). The leaves of the *N. tabacum* var. *K326* plants displayed crinkling and mild mottle symptoms, and DCSV and PMNV were detected using RT-PCR. Thus the results indicated that DCSV, PMaV1, and PMNV could effectively infect *Disporopsis* through the mechanical inoculation (Figure 9; Supplementary Figure S2).

### 3.9 Incidence of viruses infecting *Disporopsis*

The specific primer pairs (Table 1) that were designed based on the contig sequences obtained with HTS were used in RT-PCR and Sanger sequencing to detect the virus presence in 67 *Disporopsis* samples. Four viruses were detected: DCSV, the two PMaV1 isolates, DaLV and DaPTV. In addition, we detected Paris mosaic necrosis virus (PMNV), which has been reported to infect *Paris* sp. and *Polygonatum* sp. with the specific primer pair that targets its CP. The expected PCR products were recovered and sequenced (Figure 8). The results showed DaLV has the highest incidence rate and was detected in 31 out of 67 samples, followed by PMaV1 *Disporopsis* 2 isolate in 15 out of 67. PMNV was only detected in four samples from Kunming. Among the 19 samples from the Wenchuan botanical garden in the Sichuan province, only one sample was infected by DaLV, no virus in other 18 samples. All 13 samples from Kunming were infected by DaLV. Moreover, DaPTV was detected in 11 out of 67 samples, including nine samples from Kunming and two samples from Chongqing. Mixed infection was prevalent in the samples from Kunming, with 12 out of 13 being mixed infected. Six viruses including DCSV, two PMaV1 isolate, PMNV, DaLV and DaPTV were detected in 1/13. PMaV1 and DaLV were predominant viruses in the tested samples and were detected in 22 and 34 out of 67 samples, respectively (Table 4; Supplementary Table S12).

**Table 4:** Incidence of viruses infecting *Disporopsis*.

Sample host	Sample source	Sample ID	Number of detected samples	Virus						# and % of positive samples
				DCSV	PMaV1-D1	PMaV1-D2	PMNV	DaLV	DaPTV	
*Disporopsis*	Wenchuan	D-WC	19	0	0	0	0	1	0	1
	Kunming	D-KM	13	6	9	5	4	13	9	13
	Enshi	D-ES	15	3	3	4	0	0	0	12
	Chongqing	D-CQ	20	3	0	6	0	18	2	20
Total			67	12	12	15	4	32	11	46
Detectable rate				17.91%	17.91%	22.39%	5.97%	46.27%	16.42%	68.66%

## 4 Discussion

*Disporopsis* spp. have been used as medicinal plants in certain Asian countries (Sydara et al., 2014; Nguyen et al., 2019; Sarapan et al., 2023; Grabherr et al., 2011). The species belonging to the *Disporopsis* Hance genus include *D. pernyi*, *D. aspersa*, *D. fuscopicta*, *D.* *longifolia*, *D. jinfushanensis*, and *D. undulata* are native to China (Liang and Tamura, 2000). In the Yunnan and Guizhou provinces in southwest China, minority groups, such as those belonging to the Hani and Miao ethnicities, use the dried rhizome of *Disporopsis* spp. to cure pustules, cough, asthma and pneumonia (Grabherr et al., 2011; Tian, 2013). HTS provides a powerful tool for detecting the presence of known viruses and discovering novel plant viruses (Roossinck et al., 2015). So far, no viruses have been reported in *Disporopsis*. This study, used HTS to characterize the virus species in *Disporopsis pernyi* from Kumming displaying chlorotic stripe symptoms. The analysis of the virome revealed that there were five viruses, including three novel and two known virus species in this single *D. pernyi* sample. Based on the genome sequence, the three novel viruses were tentatively named *Disporopsis* chlorotic stripe virus (DCSV), *Disporopsis pernyi*-associated partitivirus (DaPTV), and *Disporopsis pernyi*-associated lispi-like virus (DaLV), belonging to *Potyvirus*, *Alphapartitivirus* and *Lispiviridae*, respectively. The two known viruses: Polygontanum mottle associated virus 1 (PMaV1) and Paris mosaic necrosis virus (PMNV), were both first reported in *Disporopsis* in this study. Two distinct strains of PMaV1, named PMaV1-*Disporopsis* 1 and PMaV1-*Disporopsis* 2, were identified within the *Disporopsis* genus. These strains were distinguished from one another by analyzing their genomic sequences.

To confirm that these five viruses were effectively capable of infecting *Disporopsis*, we homogenated samples exhibiting different viral symptoms, such as chlorotic stripes and yellow necrotic spots, and inoculated the viruses mechanically into virus-free seedlings of *D. pernyi*. The RT-PCR results confirmed that DCSV, PMaV1 and PMNV could infect *D. pernyi* using mechanical inoculation methods, while DaPTV and DaLV could not.

The phylogenetic analysis based on the RdRp amino acid sequences showed that DaLV clustered closely with maize suscal lispivirus and formed a distinct clade. A Blastp search revealed that DaLV and maize suscal lispivirus (accession number: UWX11517.1) shared the highest amino acid sequence identity (34.59%). Both DaLV and MSV shared relatively low identities (28.0%) with sequences belonging to the lispivirids and that were less than the genus-level threshold, thereby suggesting that DaLV may belong to a new genus with in *Lispiviridae* of the order *Mononegavirales*. Unfortunately, only the RdRp fragment of the DaLV genome could be obtained from the HTS data in this study. However, DaLV was detected in 32 out of 67 *Disporopsis* samples from the Yunnan province, Sichuan province, Hubei province and Chongqian city, thereby suggesting that DaLV frequently infects *Disporopsis*. Most members of this family have been identified within arthropod hosts, and these viruses are only known from their genome sequences (Li et al., 2023). In our experimental of mechanical inoculation, DaLV was not detected from the inoculated plants, indicating that DaLV might have a higher effective transmission mode in nature than artificial inoculation. Thus, further research is needed to sequence and characterize the full-length genome of DaLV, clarify other biological features, i.e., host range, vector, pathogenicity and virus-host interaction.

The genome structure of DaPTV was identical to those of alphapartitiviruses (Figure 7). Thus, we suggest that DaPTV is a member of the genus *Alphapartitivirus*. RT-PCR was carried out with primer sets (Table 1) were designed to target different regions of RNA1 and RNA2 in order to confirm the presence of this virus in *Disporopsis*, and the sequences of the RT-PCR products confirmed that they belonged to DaPTV (Figure 8). Furthermore, the phylogenetic analysis based on the RdRp and CP amino acid sequences indicated that DaPTV clustered closely with alphapartitiviruses that had been identified in plants like *Paris polyphylla* and *Poaceae liege*. DaPTV shared 87 and 78.19% amino acid sequence identities and 99–100% coverage with RdRp and CP of Paris alphapartitivirus (Table 3), which are below the species-level identity threshold of 90%. Therefore, this suggests that DaPTV may be a new alphapartitivirus. Using the specific primers targeting DaPTV, the virus was detected in 11 out of 67 *Disporopsis* samples, thereby indicating that DaPTV is prevalent in *Disporopsis*. For partivirids, bisegmented double-stranded (ds) RNA viruses without known natural vectors, their host range includes plants, fungi, and protozoa with persistent infections. The partitiviruses have hence sometimes been called cryptic viruses, or cryptoviruses, especially in the case of plant partitiviruses which generally have the asymptomatic infections in the host organisms, or have few, if any, deleterious effects on host cells (Nibert and Schiff, 2014). Plant partitiviruses were transmitted by seed (Vainio et al., 2018). Thus, further research is needed to provide the supporting evidence for partitivirus-host interactions and host effects,

An analysis on the incidence of viruses among 67 *Disporopsis* samples revealed that 46 plants out of the 67 were infected with at least one virus. PMaV1 and DaLV were the most predominantly, found viruses and were detected in 22 and 34 out of 67 samples, respectively. Mixed infections were also common, particularly in samples from Kunming.

Aside from mechanical transmission, members of the *Potyvirus* genus are usually transmitted by aphids or seed in nature (Atreya et al., 1991). The three potyviruses (DCSV, PMaV1, and PMNV) in this study may have been transmitted by aphids, agricultural practices, or contaminated seeds to *Disporopsis* from crops or weeds grown in the same or nearby fields. The viruses may also have traveled across farms and even regions on contaminated seeds. Further study is required to understand the epidemiology of these potyviruses and the importance of the transmission vectors and intermediate hosts on the virus’ pathogenicity.

This study has successfully identified and characterized three novel viruses that infect *Disoropsis* plants based on their genomic features or infectivity, and it provides a foundation for understanding the impact of these viruses on plant health and for developing future management strategies.

## Data Availability

The datasets presented in this study can be found in online repositories. The names of the repository/repositories and accession number(s) can be found at: https://www.ncbi.nlm.nih.gov/genbank/, PP691760, https://www.ncbi.nlm.nih.gov/genbank/, PP691759, https://www.ncbi.nlm.nih.gov/genbank/, PP691758, https://www.ncbi.nlm.nih.gov/genbank/, PP803564, https://www.ncbi.nlm.nih.gov/genbank/, PP803563, https://www.ncbi.nlm.nih.gov/genbank/, PP803565.

## Footnotes

^1^ https://www.ncbi.nlm.nih.gov/orffinder/

^2^ https://www.ncbi.nlm.nih.gov/Structure/cdd/wrpsb.cgi
