# Supplementary material for: Detection and discovery of plant viruses in Disporopsis through high-throughput sequencing
Source: Front Microbiol. 2024 Nov 13;15:1434554. doi: 10.3389/fmicb.2024.1434554 (PMC11599246; doi:10.3389/fmicb.2024.1434554)
Supplement: Supplementary file 3 [file Presentation_1.PPTX]

## Slide 1
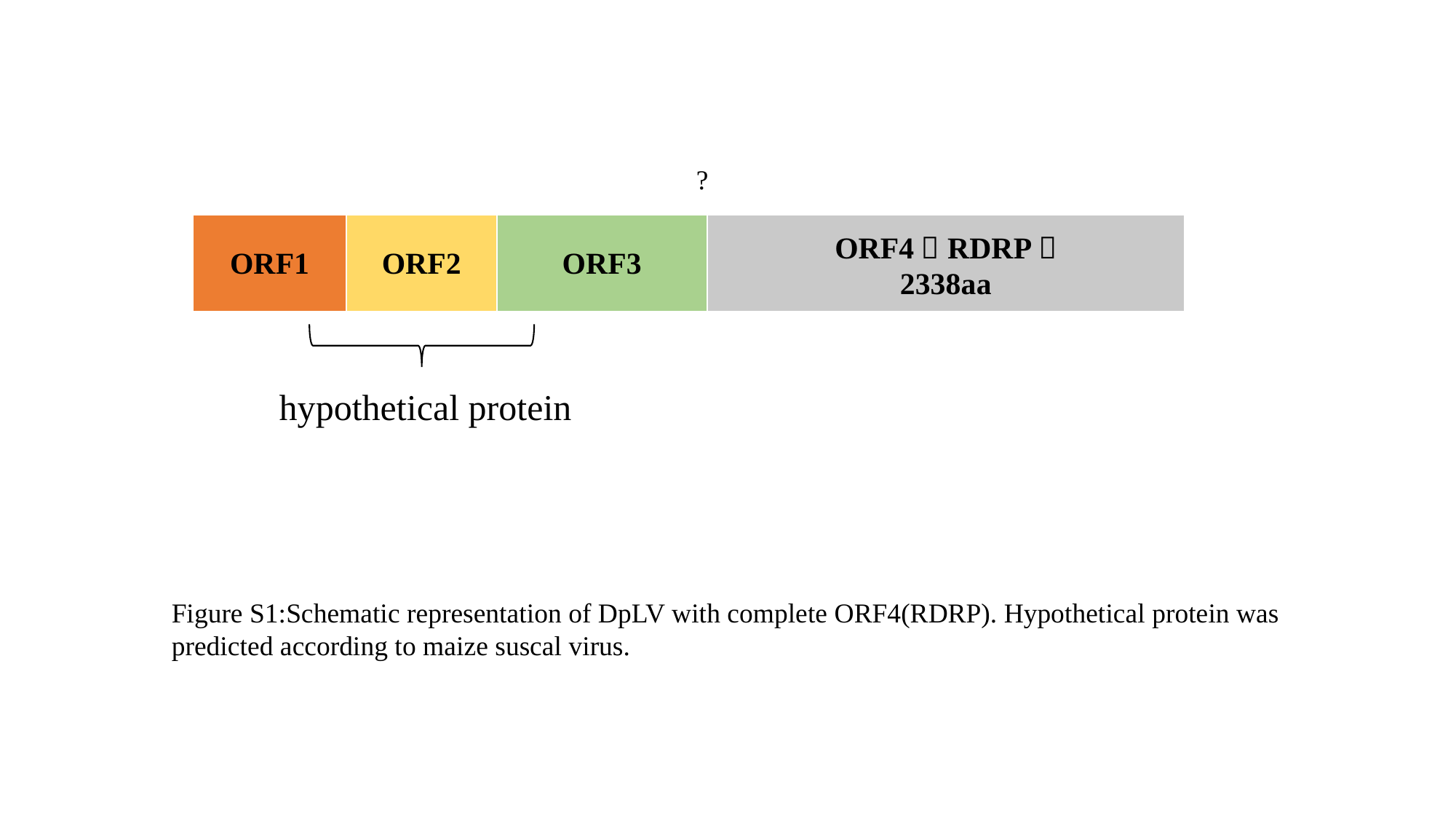

?
| ORF1 | ORF2 | ORF3 | ORF4（RDRP） 2338aa |
| --- | --- | --- | --- |
hypothetical protein
Figure S1:Schematic representation of DpLV with complete ORF4(RDRP). Hypothetical protein was predicted according to maize suscal virus.

## Slide 2
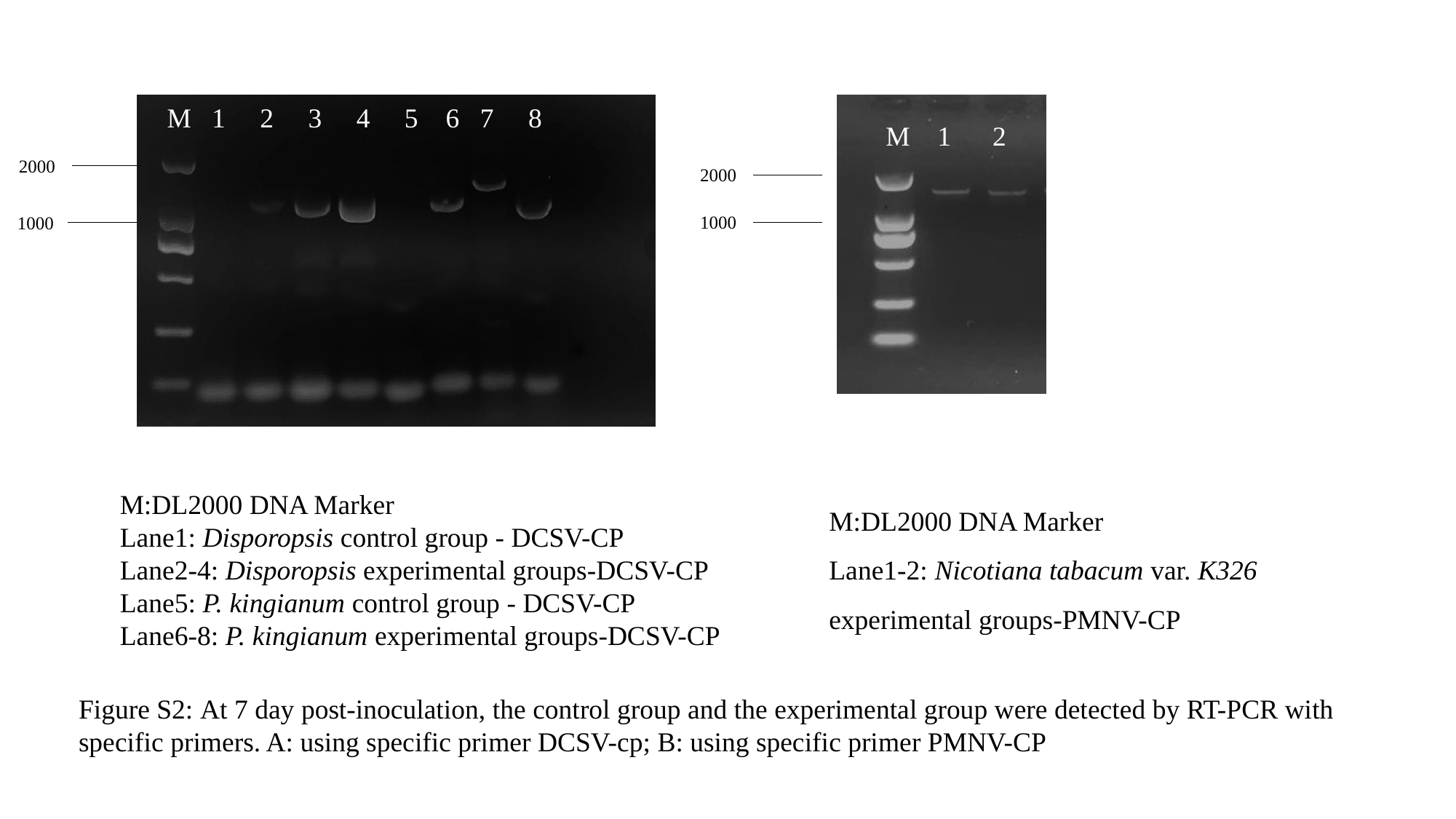

M 1 2 3 4 5 6 7 8
M 1 2
2000
2000
1000
1000
 M:DL2000 DNA Marker
 Lane1: Disporopsis control group - DCSV-CP
 Lane2-4: Disporopsis experimental groups-DCSV-CP
 Lane5: P. kingianum control group - DCSV-CP
 Lane6-8: P. kingianum experimental groups-DCSV-CP
M:DL2000 DNA Marker
Lane1-2: Nicotiana tabacum var. K326 experimental groups-PMNV-CP
Figure S2: At 7 day post-inoculation, the control group and the experimental group were detected by RT-PCR with specific primers. A: using specific primer DCSV-cp; B: using specific primer PMNV-CP
