# Supplementary figures and images for: Detection and discovery of plant viruses in Disporopsis through high-throughput sequencing
Source: Front Microbiol. 2024 Nov 13;15:1434554. doi: 10.3389/fmicb.2024.1434554 (PMC11599246; doi:10.3389/fmicb.2024.1434554)

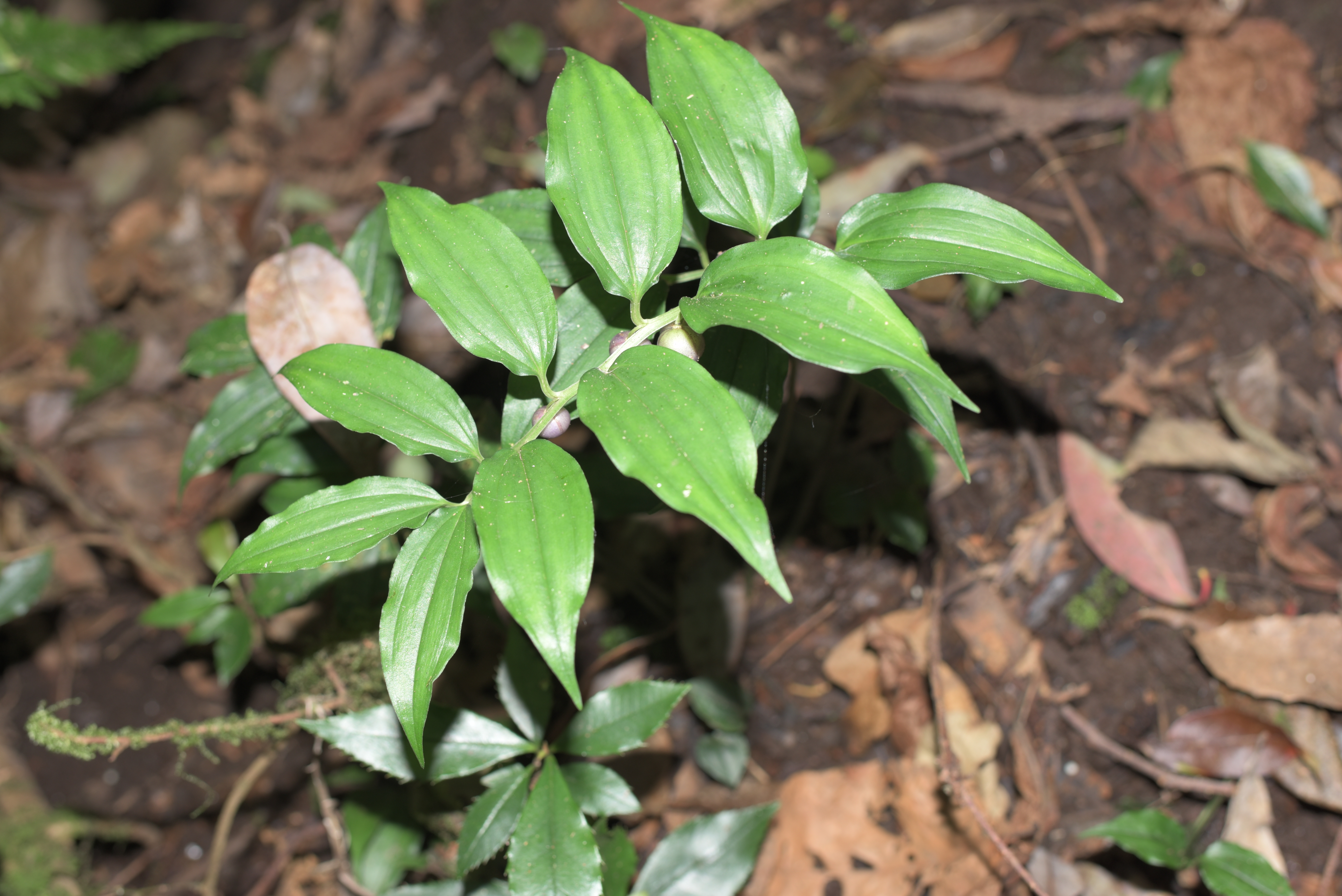

Supplement: Supplementary file 4 [file Data_Sheet_1.ZIP › Supplementary figure/Disporopsis pernyi (Hua) Diels/DSC_0924 深裂竹根七.jpg]

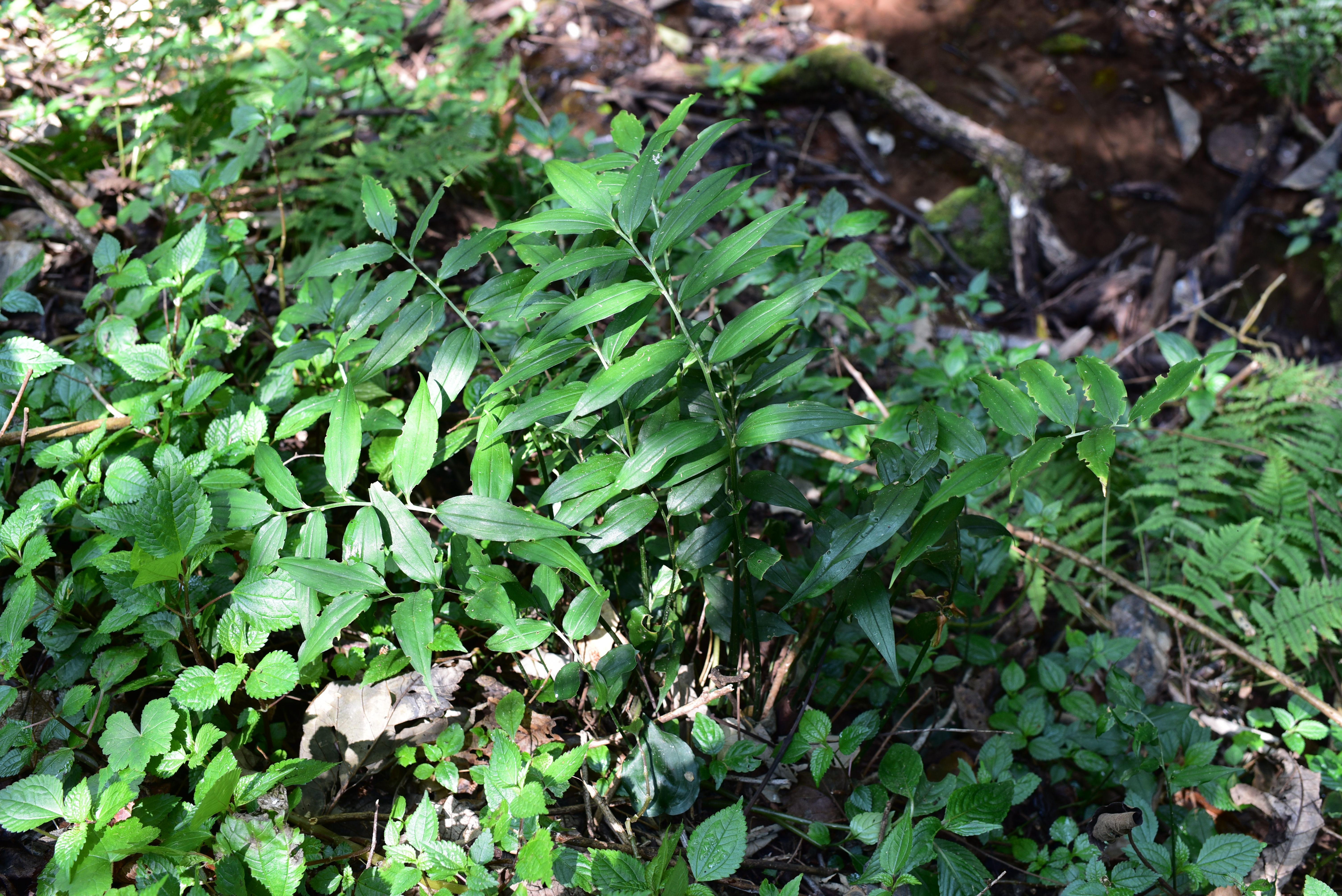

Supplement: Supplementary file 4 [file Data_Sheet_1.ZIP › Supplementary figure/Disporopsis pernyi (Hua) Diels/DSC_2551 竹根七 (新平).jpg]

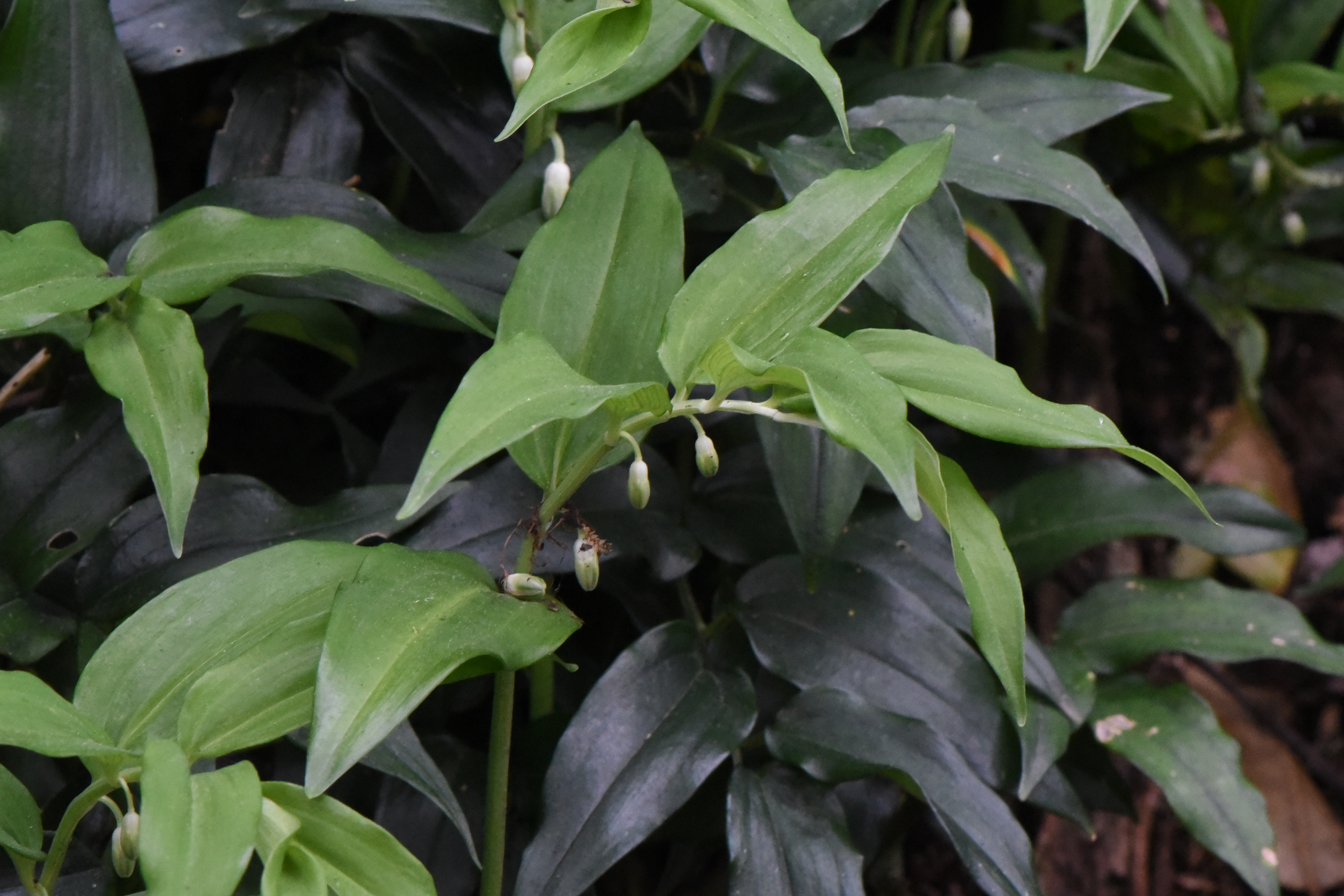

Supplement: Supplementary file 4 [file Data_Sheet_1.ZIP › Supplementary figure/Disporopsis pernyi (Hua) Diels/DSC_4747 深裂竹根七 官渡区.jpg]

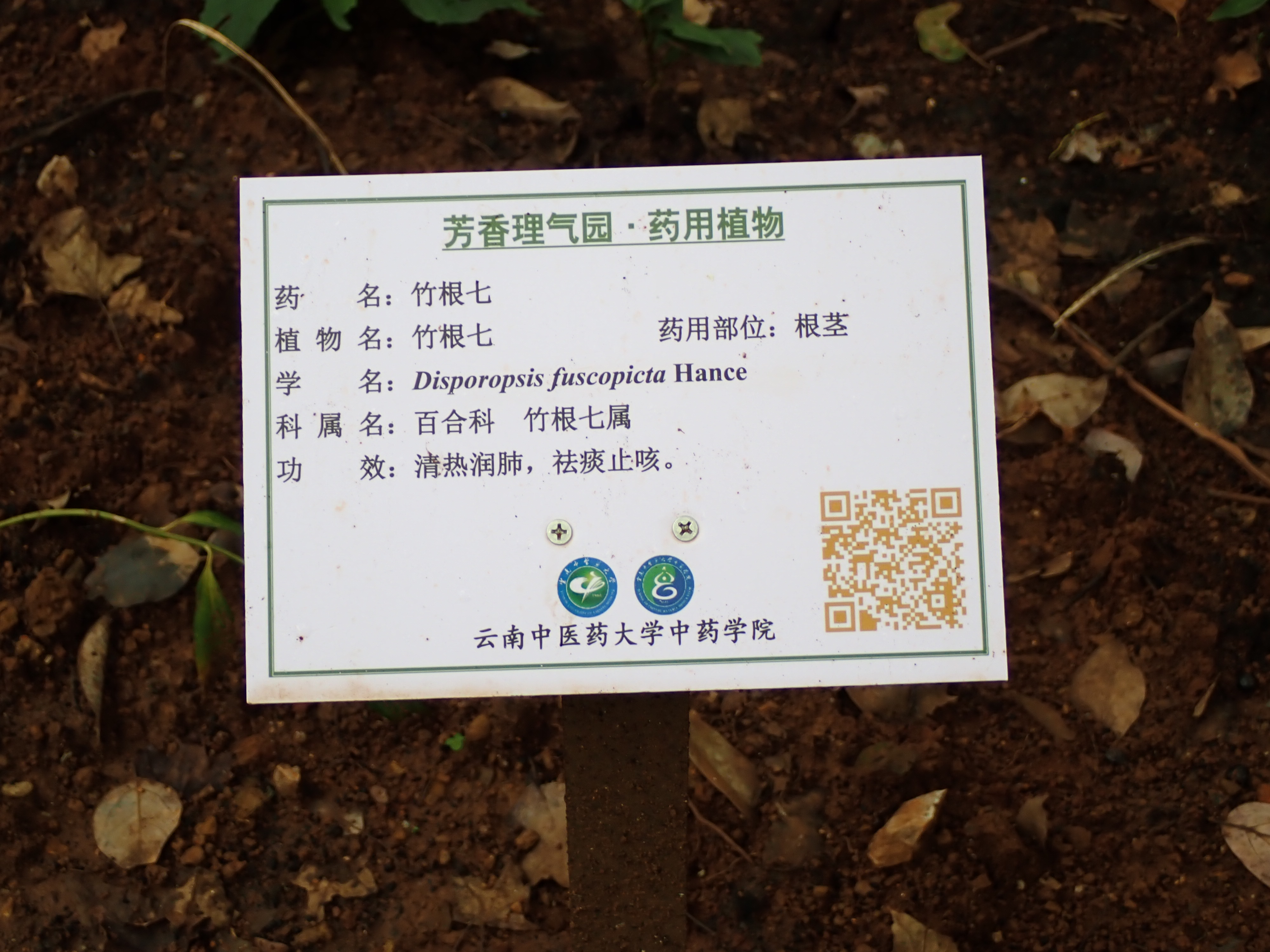

Supplement: Supplementary file 4 [file Data_Sheet_1.ZIP › Supplementary figure/Disporopsis pernyi (Hua) Diels/PA211531.JPG]

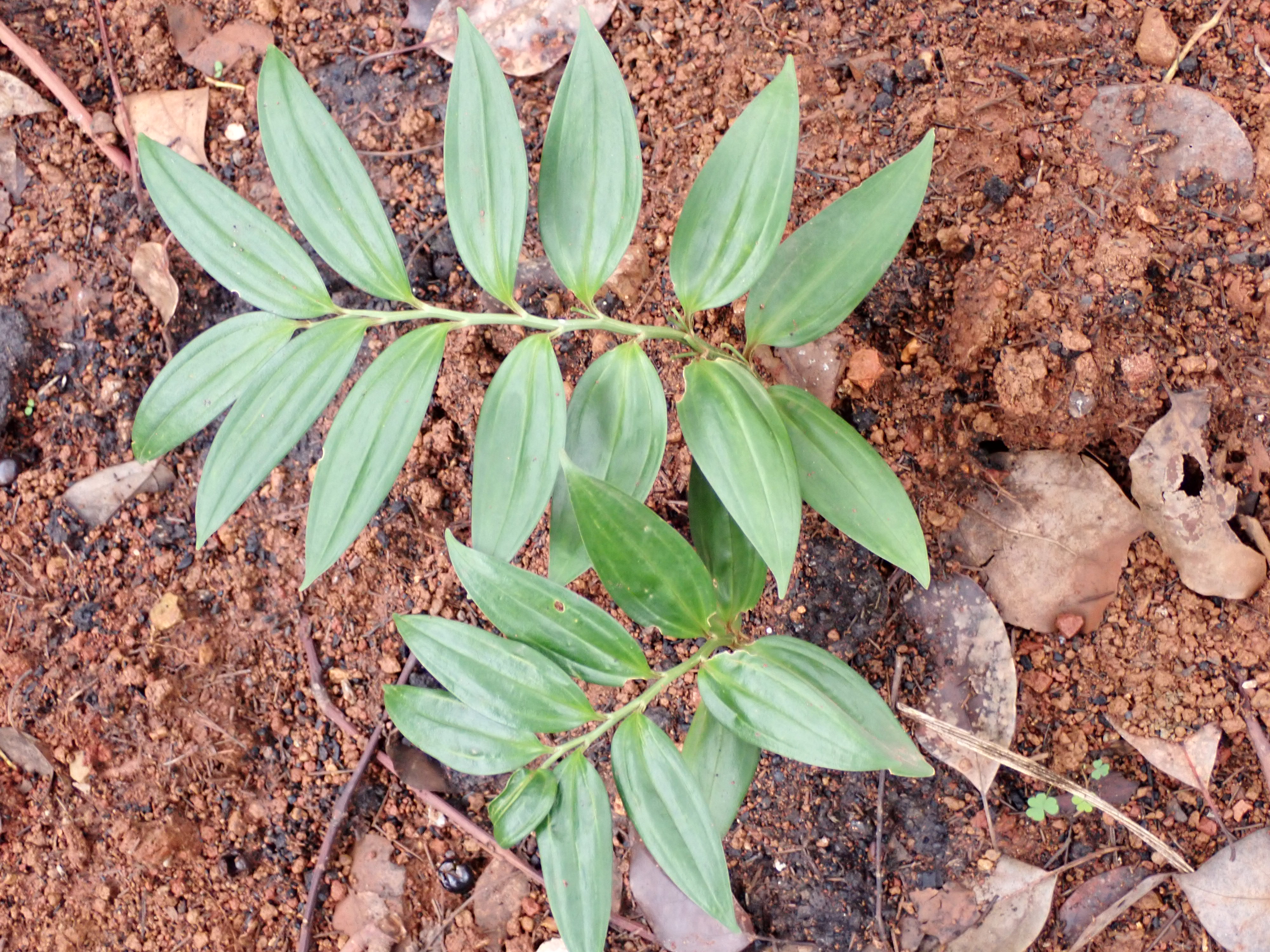

Supplement: Supplementary file 4 [file Data_Sheet_1.ZIP › Supplementary figure/Disporopsis pernyi (Hua) Diels/PA221557.JPG]

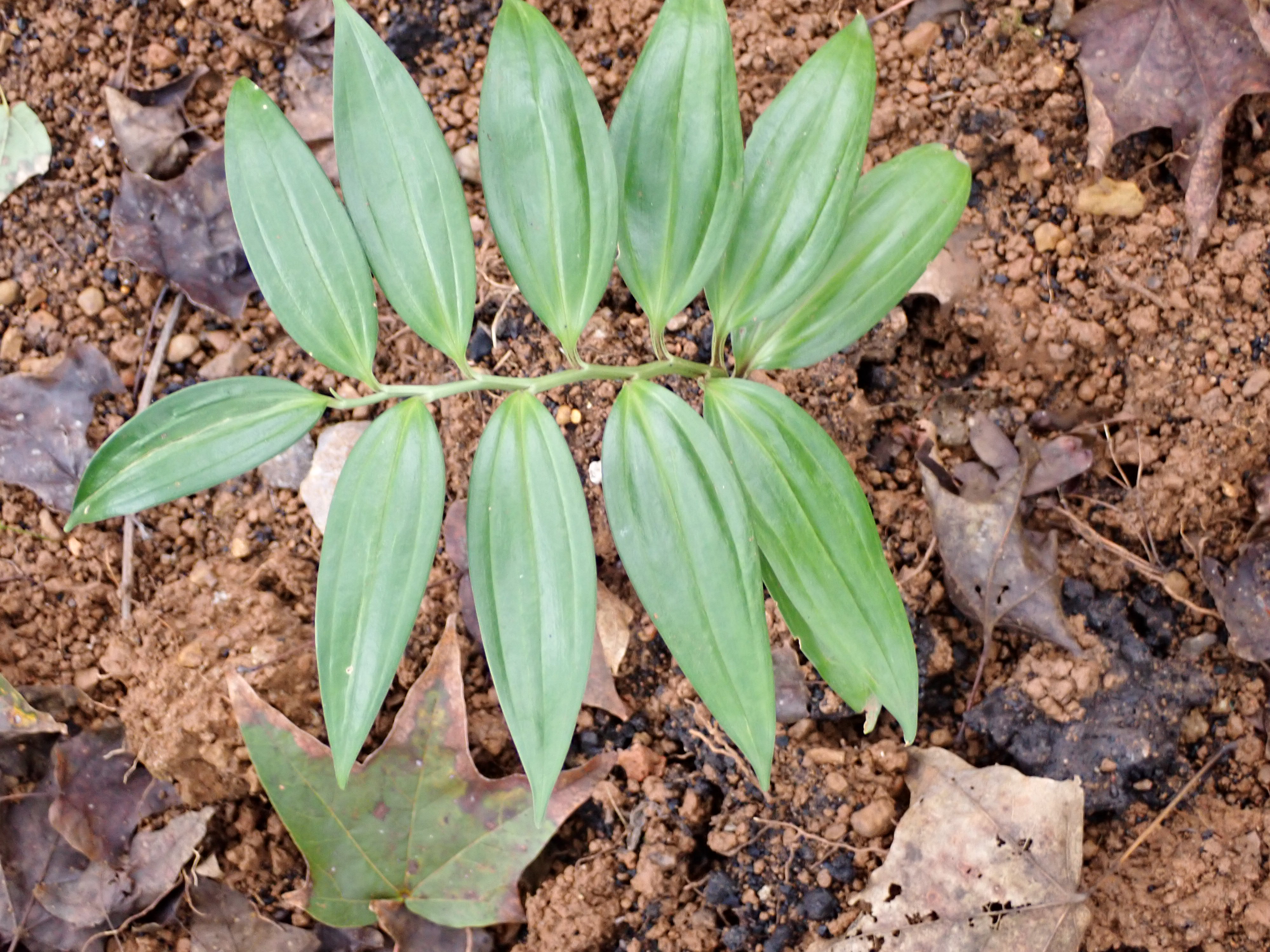

Supplement: Supplementary file 4 [file Data_Sheet_1.ZIP › Supplementary figure/Disporopsis pernyi (Hua) Diels/PA221562.JPG]
